# Fabrication of a Urea Biosensor for Real-Time Dynamic Fluid Measurement

**DOI:** 10.3390/s18082607

**Published:** 2018-08-09

**Authors:** Kyunghee Kim, Jeongeun Lee, Bo Mi Moon, Ye Been Seo, Chan Hum Park, Min Park, Gun Yong Sung

**Affiliations:** 1 Cooperative Course of Nano-Medical Device Engineering, Hallym University, Chuncheon 24252, Korea; facility.hee@gmail.com (K.K.); wjddms0906@gmail.com (J.L.); minpark@hallym.ac.kr (M.P.); 2 Department of Materials Science and Engineering, Hallym University, Chuncheon 24252, Korea; 3 Integrative Materials Research Institute, Hallym University, Chuncheon 24252, Korea; 4 Nano-Bio Regenerative Medical Institute, College of Medicine, Hallym University, Chuncheon 24252, Korea; toribom@gmail.com (B.M.M.); dpqlstj22@gmail.com (Y.B.S.); hlpch@hallym.ac.kr (C.H.P.)

**Keywords:** urea biosensor, silk fibroin, amination, flow conditions, real-time monitoring

## Abstract

In this study, a portable urea sensor that monitors the urea concentration in flow conditions was fabricated. We propose an electrochemical sensor that continually measures the urea concentration of samples flowing through it at a constant flow rate in real time. For the electrochemical sensing, a porous silk fibroin membrane with immobilized urease was mounted in a polydimethylsiloxane (PDMS) sensor housing. The fabricated urea sensor elicited linear current–concentration characteristics in the clinically significant concentration range (0.1–20 mM) based on peritoneal dialysis. The sensor maintained the linear current–concentration characteristics during operation in flow conditions.

## 1. Introduction

The kidney plays a very important role in our body. The basic function of the kidney is the discharge of physiological waste. However, it also executes numerous more biological functions, including the adjustment of physiological treatments that are indispensable for health. Other functions performed by the kidneys in the human body include the excretion of nitrogen waste and certain organic compounds; the maintenance of homeostasis and osmotic pressure; and the regulation of phosphorus, potassium, blood pressure, erythropoiesis, vitamin D synthesis, antibody expression, immune regulation, and others. More than 10 different functions, including active reabsorption processes of passive filters of body fluids of volumes of 100 L or more per day, are performed in glomeruli [1,2]. The kidney’s function cannot be restored once it deteriorates. Chronic kidney disease (CKD) is classified based on the risk and the period over which it occurs, and it is ranked in 5 stages (1 to 5) [3,4,5]. In Stage 5 of CKD, namely, end-stage renal disease (ESRD), the kidney cannot maintain homeostasis owing to the remarkable decrease in kidney function due to progressing renal failure. Inevitably, renal replacement therapy is required, including hemodialysis, peritoneal dialysis, and kidney transplantation [3,4,5,6,7,8]. Peritoneal dialysis can be managed directly from home, unlike hemodialysis. However, it is troublesome to manually replace the peritoneal dialysate (PD) four times a day, and prescribe and store more than 2 L of the infusion solution for its daily replacement [6,7,8,9]. In the case of Chronic kidney disease (CKD) patients, urea secretion and blood metabolic waste are considerably higher than the normal values because the glomerular filtration rate is less than 60 mL/min/1.73 m^2^, and because the function of the kidneys is declining [3,6,8]. Urea can be an important indicator of liver and kidney function. The standard concentration of urea in human serum is estimated to be in the range of 1.7–8.3 mM [10,11,12,13]. High urea concentrations can cause renal failure, obstructive uropathy, dehydration, shock, scald, and gastrointestinal bleeding. Conversely, low urea concentrations can cause hepatic failure, nephritic syndrome, and cachexia [10,14,15]. 

Numerous sensors destined for biological applications have been developed based on various methods, such as amperometry, potentiometry, optics, thermal measurement, piezoelectric measurements, and others [14,16,17,18,19,20,21,22]. Among the urea biosensors, the urease-based amperometric biosensor is the most extensively used because it is simple, fast, and low cost [23,24,25,26,27,28]. Urease is an enzyme that hydrolyzes one urea molecule into two ammonia and one carbon dioxide molecules. In aqueous solution, hydrolyzed ammonia molecules form ammonium ions. The rate of hydrolysis of urea by urease on the electrode surface can determine the response time of the sensor. To increase the rate of hydrolysis of urea, improved immobilization efficiency of urease ought to be achieved; the sensitivity of sensor can be greatly influenced by this improvement in immobilization efficiency. In order to increase the sensitivity of sensor, a biosensor using a nanocomposite was developed [23,24,25,26]. Another method using a cation-sensitive polyaniline (PANi) film was recently reported with a high sensitivity of 31 ± 2 mA·M^−1^·cm^−2^ [26,27,28]. A carbon electrode that can protect the defects of existing electrodes has been utilized. The carbon electrode has high activity as an electrode, and reactants, such as enzymes, can remain active on the carbon electrode [2,29]. Recently, an aminated glassy carbon electrode (GCE)-based urea sensor with immobilized enzyme on silk fibroin (SF) scaffolds was reported [11]. The GCE was functionalized with amine through electrooxidation and urease was separately immobilized on SF. This sensing platform showed improved sensitivity and rapid detection response. Furthermore, no study has been reported yet to measure the urea concentration in flow conditions. 

In this study, a portable urea sensor was fabricated using a urease-immobilized membrane for real-time monitoring of urea in PD. The screen-printed GCE was aminated through electrooxidation and used for the electrode. Urease was separately immobilized on the biopunched SF membrane for reusability. The condition for the immobilization of urease was optimized for the improvement of the sensitivity and stability of the urea biosensor. For the real-time monitoring of urea, the fluidic system was fabricated using polydimethylsiloxane (PDMS) and the whole sensor platform was assembled in 3D-printed sensor housing. To test the sensor’s real-time monitoring capacity, the linearity of the urea sensor was measured according to the various flow rates.

## 2. Materials and Methods

### 2.1. Reagent

Urease, urea, glutaraldehyde, disodium hydrogen phosphate, potassium dihydrogen phosphate, sodium chloride, and ammonium carbamate were purchased from Merck (Darmstadt, Germany). A urea assay kit was obtained from BioAssay Systems (Hayward, CA, USA). Phosphate-buffered saline (PBS) solution at a concentration of 20 mM was prepared by mixing 20 mM disodium hydrogen phosphate, 20 mM potassium dihydrogen phosphate, and 20 mM sodium chloride, and its pH was adjusted to 7.4. PBS was used as the supporting electrolyte during all the electrochemical measurements. All solutions were prepared using 18.2 MΩ purified water produced with a water purification system (Simplicity, Millipore, Burlington, MA, USA). All glassware and polyethylene materials were rinsed with ultrapure water and dried before use. Before each experiment, all stock solutions were freshly prepared.

### 2.2. Preparation of Porous Silk Fibroin Membrane and Urease Immobilization

**Figure 1:**
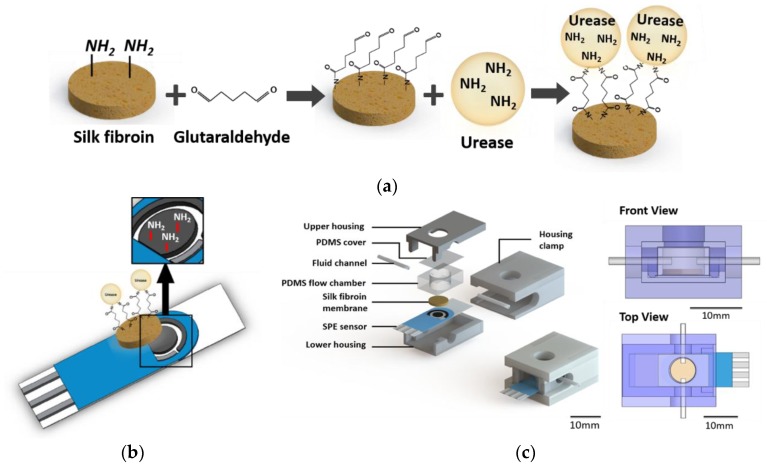
Schematic diagram of (**a**) the principle of immobilization of urease on the silk fibroin (SF) membrane, (**b**) the amination of the surface of the carbon electrode, and (**c**) the configuration of the sensor system.

Porous SF scaffolds were produced by a salt leaching method to form a porous structure [11,29,30]. Circular SF membrane discs (diameter: 8 mm, thickness: 2.5 mm) were prepared by using a bio-punch. Urease was immobilized on the surface of porous SF membranes via crosslinking of proteins by glutaraldehyde. As shown in Figure 1a, the primary amine group of SF membrane was modified to an aldehyde group by the treatment of 10% glutaraldehyde solution with PBS at 25 °C for 3 h. After washing, the modified SF membrane was reacted with a urease solution at 4 °C for 24 h. The urease-treated SF membrane was washed with PBS and stored at 4 °C in PBS.

### 2.3. Urease Activity Assay

A commercial urea assay kit was used to measure the urea concentration. The urease-immobilized SF membrane was reacted with 16.67 mM urea for 1 h at 25 °C by shaking (*n* = 3). After the hydrolysis of urea by the urease-immobilized SF membrane, 5 μL of the reacted urea solution was transferred into a 96-well microplate. Subsequently, 200 μL of the phthalaldehyde reagent in the kit was added to initiate the colorimetric reaction. After 20 min of reaction time, the optical density (OD) was measured at a wavelength of 520 nm for the quantification of the remaining urea. For the calculation of the urea concentration, 50 mg/dL of urea was used as a standard solution, while PBS was regarded as a blank sample. 

### 2.4. Amination of Screen-Printed Carbon Electrodes

In this research, a three-electrode electrochemical system with screen-printed carbon (DropSens, Llanera, Spain) was used after the amination. As shown in Figure 1b, the working and counter electrode was screen-printed carbon, and the reference electrode was manufactured with silver. The diameter of the working electrode was 4 mm. The surface of the electrode was thoroughly washed with water or ethanol, and the surface of the working electrode was then modified according to methods reported in the literature [11,15]. Briefly, the electrode was dipped in 0.5 M ammonium carbamate solution, and amination was performed using cyclic voltammetry (CV). The sweeping potential and rate of CV were respectively set at values within the range of 0.5 to 1.2 V, and at 20 mV/s, using 50 cycles [11]. After amination, the electrode was washed with deionized water (DW).

### 2.5. Fabrication of Urea Biosensor Flow System

#### 2.5.1. Cylindrical Microfluidic PDMS Chamber Fabrication

PDMS was selected for the fabrication of the cylindrical microfluidic chamber owing to its biocompatible properties and ease of processing. The PDMS agent was solidified in a Si wafer adhered to a plastic frame for 4 h at 80 °C. The solidified PDMS was then removed from the frame and was cut to 10.20 mm in width, 14 mm in length, and 5.43 mm in height (Figure 1c). The cylindrical microfluidic chamber with an inner diameter of 8 mm was drilled with a bio-punch.

#### 2.5.2. Housing Design

As shown in Figure 1c, the overall structure of the sensor housing consisted of a lower housing, an upper housing, a housing clamp, a PDMS microfluidic chamber, and a membrane. For the housing fabrication, the extensively used acrylonitile poly-butadiene styrene (ABS) filaments were selected because of their low price, excellent adhesiveness, and low melting point. Furthermore, this material has the advantage that its treatment is possible with sandpaper, and it is easy to manufacture. Using ABS filaments in the 3D printer (CUBICON, Seongnam-si, Korea), the lower housing (16.20 mm width, 27.0 mm length, 5.47 mm height), upper housing (16.20 mm width, 27.0 mm length, 3.48 mm height), and housing clamp (22.20 mm width, 30 mm width, 14.95 mm height) were implemented. Owing to the material properties of ABS, high-speed cooling may cause bending at the time of production. To prevent bending, the board of the 3D printer was thoroughly preheated and kept at a high temperature until the output material was formed.

#### 2.5.3. Flow System Configuration

For the real-time monitoring of urea in conditions of flow, a single-flow system was constructed to detect the urea concentration at the flow. The fabricated PDMS sensor flow cell was connected with the liquid transfer tube, and an open circuit was created through the fluidics. The flow rate can be set to the desired operating condition with a peristaltic pump (ISMATEC, Wertheim, Germany) using an open circuit that connects to the tube in the PDMS chamber inlet side, and to a waste bottle in the outlet side. Potentiostats (DropSens, Llanera, Spain) were connected to the electrode installed in the sensor’s housing so that current was generated from the hydrolysis of urea by urease:

$$\mathrm{Urea}+3\;H_{2}O\;\overset{\mathrm{Urease}}{\to }\;2\;{\mathrm{NH}}_{4}^{+}+{\mathrm{OH}}^{-}+{\mathrm{HCO}}_{3}^{-}.$$

The generated current was converted into a digital signal to allow the measured data to be saved and transferred. Before the measurement, the urea sensor system was stabilized by flushing it using PBS for 20 min.

## 3. Results and Discussion

### 3.1. Urease Immobilization on SF Membrane

**Figure 2:**
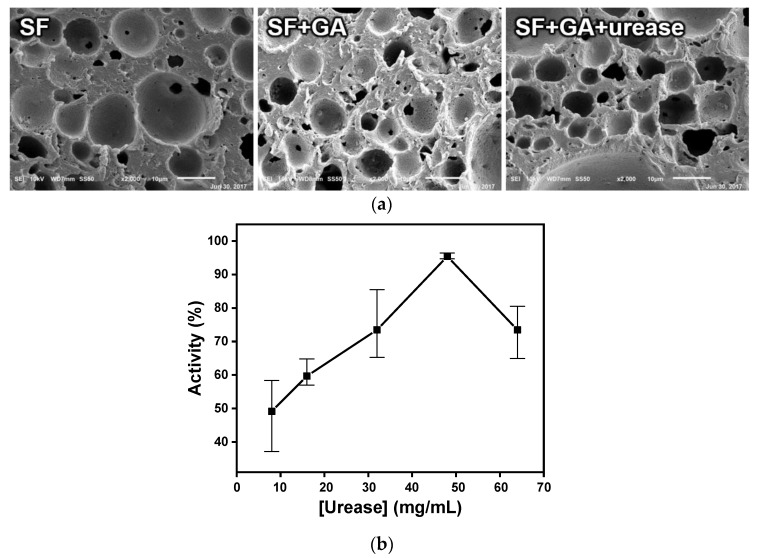
(**a**) The microstructural topography changes during the urease immobilization process on porous SF membrane (GA: glutaraldehyde). (**b**) Urease immobilization efficiency according to the treated urease concentration.

In this study, urease was not immobilized directly to the sensor’s electrode but was immobilized on the SF membrane so that the hydrolysis of urea was indirectly supplied to the sensor electrode. Since urease is an enzyme, it is very difficult to manage its activity based on environmental conditions, such as temperature and pH. There are numerous disadvantages when urease is immobilized directly to the sensor’s electrode in that the lifetime of the sensor becomes very short, reuse of the enzyme is impossible, and the immobilization efficiency is unstable [11,31,32]. In contrast, a urease-immobilized membrane-based biosensor is expected to overcome these limitations. To increase the urease immobilization efficiency of the urea biosensor, SF, an Food and Drug Administration (FDA)-approved natural product from the *Bombyx mori* silkworm, was used. SF has been extensively used in various biological fields owing to its high tensile strength, adjustable biodegradability, low antigenicity, and non-inflammatory properties [11,18,29,30]. For the immobilization of urea on the porous SF membrane, glutaraldehyde was selected as the crosslinking agent. Since SF is a protein containing numerous amine groups, the treatment of glutaraldehyde in SF resulted in an amide bonding between the amine on SF and aldehyde in glutaraldehyde, and the amine on the surface of the SF membrane was modified into aldehyde (Figure 1a). Urea can then be immobilized on the SF membrane via another amide bonding between that aldehyde and the amine in urease. This immobilization achieved by the crosslinking of urease with the SF membrane increases the resistance to chemical and biological degradation. As shown in Figure 2a, the surface morphologies during the crosslinking process were analyzed with an electron microscope. An arbitrary SF porous structure was found when the membrane was prepared using the salt leaching method. This macroporous structure with diameters ranging from 2 to 30 μm was formed using salt powders. Following a sequential treatment of glutaraldehyde and urease, the surface morphology appeared slightly disturbed, but the macroporous structures were still effective. From these results, it was confirmed that the porous structure of the SF membrane was not affected during the urease immobilization process. 

A urea assay was used to confirm the immobilization efficiency of urease on the SF membrane. After the treatment of various concentrations of urease, a urease activity test was performed by using a commercial urea assay kit. As shown in Figure 2b, urease concentrations of 8, 16, 32, 48, and 64 mg/mL were used to treat the aldehyde-functionalized SF membrane. The removal rate represented the activity of immobilized urease on the SF membrane. By increasing the concentration of treated urease, the urea removal rate was increased, and the maximal urea removal rate achieved at 48 mg/mL was approximately 95%. The removal rate then decreased when the treated concentration of urease was higher than 48 mg/mL. At concentrations lower than 48 mg/mL, the amount of treated urease was less than the total capacity of the SF substrate, and a urease concentration of 48 mg/mL yielded maximum efficiency. At concentrations above 48 mg/mL, highly immobilized urease molecules were thought to affect each other, and decreased the degrees-of-freedom so that the immobilization efficiency was reduced. Based on these results, the concentration of urease for real-time urease monitoring was optimized to be 48 mg/mL.

### 3.2. Modification of Electrode’s Surface

**Figure 3:**
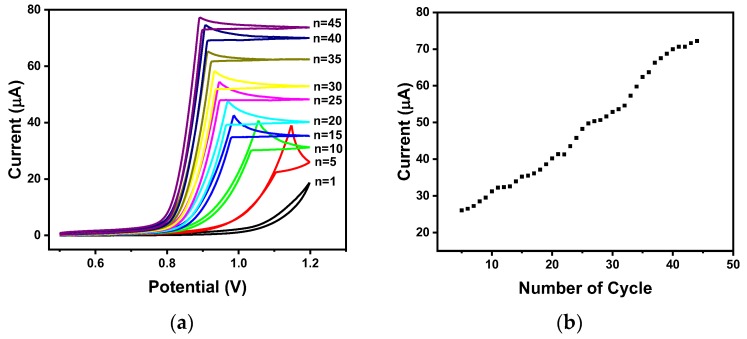
The amination of carbon electrode with 0.5 M ammonium carbamate. (**a**) CV voltammogram and (**b**) its maximal oxidation currents by cycles.

It was confirmed that the sensitivity and stability of carbon electrodes for performing electrochemical measurements were increased after lengthy use [11]. Urease hydrolyzes urea to produce ammonium ions and carbon dioxide. During this hydrolysis reaction, carbamic acid is produced as an intermediate and captures one electron from the electrode’s surface to form carbamic acid radicals. This process is repeated continually, and carbamic acid radicals accumulate. These accumulated carbamic acid radicals react with each other to form carbamic acid dimers. At the same time, a carbon–nitrogen bond is formed between the carbon on the surface of the carbon electrode and the nitrogen in the carbamic acid radical. Based on the decarboxylation, an amine group can be formed on the surface of the carbon electrode. This amine-functionalized electrode is estimated to increase the sensitivity and stability of the sensor [11]. Therefore, for real-time monitoring at flow conditions, the amination of the surface of the electrode is an essential step to increase the sensitivity and the stability of the sensor. In this study, the surface of the carbon electrode was aminated by CV using ammonium carbamate. When the ammonium carbamate molecule was hydrolyzed, carbamic acid was produced, and carbamic acid radicals were also generated using the applied potential. The surface of the carbon electrode was then aminated by decarboxylation. As shown in Figure 3a, the electrode was aminated using 0.5 M ammonium carbamate solution via CV in a voltage range of 0.5 to 1.2 V with the use of 45 cycles. The data showed that the oxidation current value greatly increases during the first 40 cycles, and the rate of increase becomes smaller after 40 cycles. From these data, it was deduced that the surface of the carbon electrode was successfully aminated after 40 cycles of CV.

### 3.3. Real-Time Monitoring of Urea Concentration

**Figure 4:**
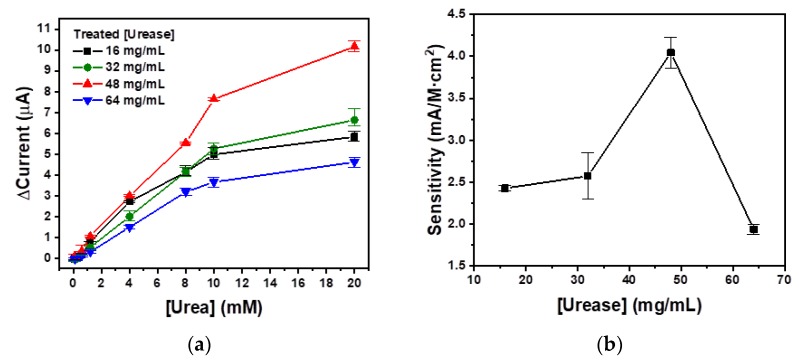
(**a**) Chronoamperometric urea sensing and (**b**) sensitivities with various concentrations of urease-treated SF membranes for the optimization of urease concentration.

The urea concentration in healthy controls ranged from 1.7 mM to 8.3 mM, but its value was much higher in ESRD patients. After the peritoneal dialysis, the urea concentration of the dialysate should be decreased. For real-time monitoring of urea in the PD, measurement was performed over a wide range of concentrations with a maximum concentration of 20 mM. In prior, conventional research studies, only a few urea sensors have been implemented that have elicited stable measurements within a large-scale range. Additionally, these sensors had been studied under static conditions; thus, a real-time monitoring urea sensor is required to be developed for an artificial kidney system. Furthermore, the sensitivity of sensors implemented previously was greatly decreased in conditions of flow environments. Therefore, high sensitivity is also a key component for the real-time monitoring of PD. In this study, chronoamperometry, which is a current measurement method over time at a constant, optimal voltage of 1.1 V, was used. In Figure 2b, the maximum urease activity used for the immobilization on the SF membrane was observed to be 48 mg/mL. For the confirmation of the correlation between immobilized urease activity and the chronoamperometric urea sensing ability, urea sensing tests were performed with urease concentrations of 16, 32, 48, and 64 mg/mL in the presence of a flow of 0.5 mL/min, as shown in Figure 4. The concentrations of urea solution were sequentially increased from 0.1 mM to 20 mM, and each urea solution was treated for 20 min. The average current values of each urea concentration were calculated and are plotted in Figure 4a. The current value generated for a 48 mg/mL urease-treated SF membrane (▲) yields the highest values when the urea concentrations are higher than 1.2 mM. All urease-treated SF membranes show good linearity when the urea concentrations are below 10 mM. The slopes of the urease-treated SF membranes at the concentrations of 16, 32, 48, and 64 mg/mL within this urea concentration range were calculated to be 0.51, 0.54, 0.74, and 0.38, respectively. This means that urease-treated SF membrane at a concentration of 48 mg/mL yielded the most sensitive current change according to the change of urea concentration. As shown in Figure 4b, the sensitivities of the urease-treated SF membranes at concentrations of 16, 32, 48, and 64 mg/mL were calculated to be 2.34, 2.63, 4.03, and 1.86 mA/M·cm^2^, respectively. Based on the elicited results, the optimal urease treatment concentration for the chronoamperometric biosensor was 48 mg/mL. Furthermore, the maximal slopes and sensitivities of the urease-treated SF membrane at a concentration of 48 mg/mL correspond perfectly with the urease immobilization efficiency in Figure 2b. This means that urease immobilization with high efficiency also leads to high efficiencies for chronoamperometric urea biosensing. 

**Figure 5:**
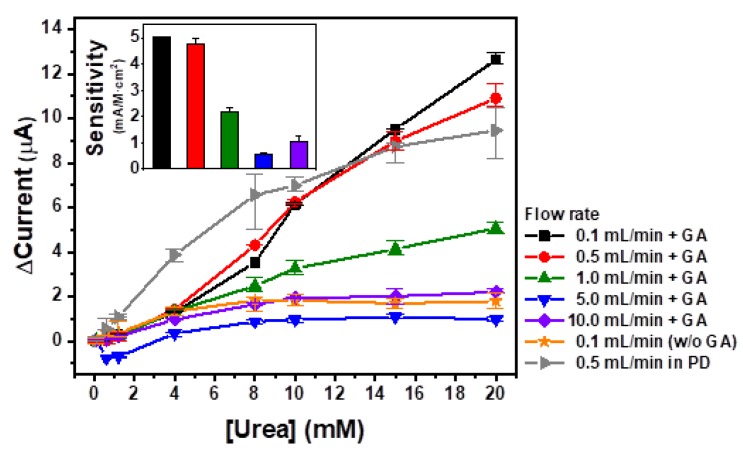
The real-time monitoring of urea concentration with various flow rates by the urease-immobilized SF-membrane-based urea biosensor.

The immobilized urease on the SF membrane-based urea sensor was tested at various flow rate conditions. The SF membrane treated with 48 mg/mL urease was used for urea biosensing, and each of the concentrations of the urea solutions at flow rates of 0.1, 0.5, 1.0, 5.0, and 10.0 mL/min, were measured for 20 min using chronoamperometry. The average current values of each urea concentration were calculated and plotted in Figure 5. As shown in this figure, 0.5 mL/min of flow rate (▼) yields the highest current values at urea concentrations lower than 15 mM, while a flow rate of 0.1 mL/min (◆) generates the highest current value at the maximum urea concentration (20 mM). In cases of higher flow rates (>1.0 mL/min), the maximum current values were lower than 5 μA. When all data were fitted with linear regressions, slope values were calculated to be 0.65, 0.59, 0.27, 0.08, and 0.12 corresponding to the flow rates of 0.1, 0.5, 1.0, 5.0, and 10.0 mL/min, respectively. Conversely, higher flow rates, such as 5.0 (●) and 10.0 (■) mL/min showed relatively lower slopes. This means that at flow rate conditions less than 1.0 mL/min, the feasibility of the urease-immobilized SF-based urea sensor was confirmed for real-time urea monitoring, while the reliability rapidly decreased at increased flow rates at values higher than 5.0 mL/min. In the case of a flow rate of 1.0 mL/min (▲), even if the linearity was significantly reliable, the slope was less than half the slope value obtained at a flow rate of 0.5 mL/min (▼). The sensitivities of the flow rates at 0.1, 0.5, 1.0, 5.0, and 10.0 mL/min were calculated to be 5.02, 4.35, 1.99, 0.70, and 0.85 mA/M·cm^2^, respectively (Figure 5, insert). These sensitivities support the reliability of the measurements of the immobilized urease on the SF-membrane-based urea sensor over a broad flow rate range. For the confirmation of the immobilization of urease on the SF membrane by chemical cross-linking, 48 mg/mL urease-treated SF membrane treated without glutaraldehyde (GA) (★) was tested. In this case, urease can attach onto the SF membrane by only physical adsorption. The maximum current value was obtained at 10 mM urea and it was 3.4-fold lower than that of chemical cross-linked SF membrane. The current values were decreased when the urea concentration was higher than 10 mM. This means that the immobilization efficiency of physical adsorption is much lower than that of chemical cross-linking, so GA cross-linking is essential for the immobilization of urease on the SF membrane. Urea samples were prepared by spiking urea in PBS and measured using the fabricated urea sensor. Unlike PBS, clinical samples such as peritoneal fluid and blood might contain interfering species. Therefore, the relevance of measurements in physiological samples could be considered. Urease is the urea-hydrolyzing enzyme that can recognize urea specifically in a physiological complex. Various studies have reported that urease-based sensors stably perform without influence by interfering species such as thiourea, creatinine, glucose, uric acid, ascorbic acid, glycine, cations, anions, and others [33,34,35]. The fabricated urea sensor was based on a urease-immobilized SF membrane, so it was expected to stably perform in clinical samples. For the test of a real sample, PD with 20 mM urea concentration and its diluent in PBS (►) were measured using a flow rate of 0.5 mL/min. The current values were increased when the urea concentrations were increased and the sensitivity was calculated to be 4.62 mA/M·cm^2^. These data were comparable with the data from urea in PBS at the same flow rate. This means that the urease-immobilized SF-based urea sensor was confirmed to be feasible for real-time urea monitoring in PD at a flow condition. To test reproducibility and accuracy, 20 mM urea at a flow rate of 0.1 mL was measured 3 times using 48 mg/mL urease-treated SF membrane. For the determination of reproducibility, the relative standard deviation was used and the reproducibility was calculated to be 11.4%. The accuracy was calculated to be 97.7%. The reproducibility at less than 15% and accuracy at nearly 100% indicate high reliability of the developed portable urea biosensor. Based on these results and optimizations, it was shown that the immobilized urease on the SF-membrane-based urea sensor is feasible for real-time monitoring of urea concentrations at flow rates less than 1.0 mL/min.

In this research, hydrolyzing urease was immobilized on separated porous SF membranes by chemical cross-linking. These membranes were subsequently utilized for real-time monitoring of urea concentrations using chronoamperometry. After the optimization of the concentration of treated urease, amination cycles, and applied potentials, urea biosensing was performed at various flow conditions. From the elicited results, the immobilized urease on SF-membrane-based urea biosensors was confirmed to be feasible for real-time monitoring of urea concentrations in flow conditions, and was shown to be capable of being used in portable artificial kidney systems for the monitoring of urea in PD.

## 4. Conclusions

The flow system consisted of a PDMS flow chamber, and a screen-printed carbon electrode was designed for the real-time monitoring of urea in flow conditions. This fluidic system was easy to fabricate and achieved cost reduction and downsizing for use as a portable urea sensor. For the sensitive measurement of urea concentration in flow conditions, a urease-immobilized SF-membrane-based urea sensor was fabricated. The various flow conditions were tested, and were confirmed to be feasible for real-time monitoring at flow rates of from 0.1 mL/min to 0.5 mL/min. This urea sensor, which measures the urea concentration in flow conditions, has a strong advantage in applications to various fields such as portable peritoneal dialysis and hemodialysis systems. At flow rates higher than 1.0 mL/min, the sensitivity of the urea biosensor was not sufficient for real-time monitoring, and modifications to the urease-immobilizing membrane should thus be studied further. This urea biosensor could constitute one of the key components of a wearable artificial kidney system for the monitoring of urea in PD of ESRD patients.
